# Prediction of the success of orthodontic treatment of impacted maxillary canines using panoramic radiography parameters: a retrospective cross-sectional study

**DOI:** 10.1186/s12903-024-05343-x

**Published:** 2024-12-24

**Authors:** Yusuf Ömer Güllü, Fethiye Çakmak Özlü

**Affiliations:** Faculty of Dentistry, Department of Orthodontics, Ondokuz Mayıs University, Atakum, Samsun, 55139 Turkey

**Keywords:** Impacted maxillary canine, traction duration, success of treatment, orthodontics

## Abstract

**Background:**

This retrospective study aimed to investigate the relationships between the radiographic features of impacted maxillary canines (IMCs) and traction duration and the factors affecting treatment success.

**Methods:**

Pre-treatment panoramic radiographs and patient records of 121 consecutive patients with IMCs were analyzed. The measurements included the angle of the IMC with the midline (α-angle), the horizontal position of the IMC relative to the adjacent teeth (S-Sector), the distance from the IMC to the occlusal plane (d-distance), the apex position of the IMC (A), and the vertical height of the IMC relative to the adjacent lateral tooth (V). The measurements were repeated 1 week later. The intraclass correlation coefficient was used to evaluate the relationship between two measurements. Binary logistic regression was performed to determine the factors affecting treatment success. Linear regression was conducted to determine the relationships between traction duration and other parameters. Receiver operating characteristic (ROC) curve analysis was performed to determine the α-angle and the d-distance cutoff values for treatment success. The significance level was set at *p* < 0.05.

**Results:**

The buccally IMCs were mostly located in sectors 1–2, whereas the palatally IMCs were mostly located in sectors 3-4-5. The effects of the parameters on the treatment success were significant according to the logistic regression of age (*p* = 0.003), d-distance (*p* = 0.002), and α-angle (*p* = 0.001). Linear regression analysis revealed that traction duration was statistically significant (*p* < 0.001).

**Conclusion:**

According to the results of this retrospective study, the α-angle, d-distance, and sector can be used to predict the buccopalatal position of the IMC. The patient’s age, d-distance, and α-angle affected the treatment success. The patient’s age, d-distance, and the sector of the impacted canine affected the traction duration.

**Clinical trial number:**

Not applicable.

## Introduction

Canine teeth are very important for the dental arch in terms of function and aesthetics. Canine impaction may cause deterioration of the function and aesthetic integrity of the dental arch. Maxillary canine teeth are the most commonly impacted teeth following the lower third molars [1], and their prevalence varies between 0.27% and 5.9% depending on the population [2]. The impact of the maxillary canine is 85% palatal and 15% buccal [3]. Impacted maxillary canines (IMCs) have been shown to occur twice as commonly in females as in males, and only 8% of IMCs are bilateral [4].

To date, many studies have reported treatments aimed at preventing maxillary canine teeth with ectopic eruption from becoming impacted and bringing them into their correct positions, especially during the mixed dentition period [5–11]. The first study on the eruption probability of maxillary canines with an ectopic eruption path was published by Ericson and Kurol [5]. Many studies [12–17] have proposed methods for predicting the probability of ectopic eruption of maxillary canines, such as the method described by Ericson and Kurol. However, when a patient’s dental development progresses and the maxillary canine is impacted, orthodontic traction should be applied after surgical exposure. Therefore, the methods of prediction proposed for preventive treatment have begun to be used to evaluate the prognosis of orthodontic traction. Ericson and Kurol measured the sector position, the angle with the midline, and the distance from the occlusal plane of maxillary canines on panoramic radiographs. These measurements have subsequently become popular in relation to outcomes such as the prediction of impacted teeth [18–20], duration of orthodontic treatment [21–25], risk of root resorption [26–30] of adjacent teeth, periodontal [31, 32] and esthetic [33] outcomes, orthodontic malocclusion [34], and dental anomalies [35].

In the literature, studies [27, 36, 37] evaluating the radiographic position of IMCs have focused mostly on treatment duration. Few studies [33, 38, 39] have investigated only the factors affecting treatment success by comparing treatment results.

Reliable predictions of the treatment success and treatment duration for impacted canines will help provide accurate information for both the clinician’s treatment planning and the patient’s decision-making process. Therefore, this study aimed to examine the relationship between pre-treatment radiographic measurements and the treatment outcomes of patients with IMCs via certain prediction methods. The alternative (H1) hypothesis of our study was ‘The success of orthodontic treatment of IMCs and the traction duration depend on the measurement parameters on panoramic radiographs’.

## Methods

### Study design and sample

The pre-treatment records of 1027 patients who were treated for impacted teeth at Ondokuz Mayıs University, Faculty of Dentistry, Department of Orthodontics, between 2010 and 2023 were retrospectively examined to determine the position of the IMC. The final sample included 121 patients (134 IMCs) according to the inclusion and exclusion criteria. Approval for this retrospective study was granted by Ondokuz Mayıs University Faculty of Medicine Clinical Research Ethics Committee in Samsun, Turkey (date of 03.24.2023 – approval number: 2022/378). This retrospective cross-sectional study followed the principles of the Declaration of Helsinki. All patients (and parents of patients under 18 years of age) signed a written informed consent form allowing their data and radiological findings to be used for future research.

### Criteria

The inclusion criteria were as follows: (1) patients who had been treated for unilaterally or bilaterally intraosseous IMC; (2) patients whose pre-treatment panoramic radiographs of appropriate quality were available; and (3) patients whose clinical records were written by the treating orthodontist.

The exclusion criteria were as follows: (1) patients diagnosed with any systemic disease that delays or prevents tooth eruption; (2) patients with transposition anomalies or tooth agenesis in the maxillary canine region; (3) patients with excessive root resorption of the impacted canine or adjacent teeth; (4) patients with any local factor preventing eruption in the maxillary canine region; (5) patients with craniofacial/congenital anomalies such as cleft lip/palate and cleidocranial dysostosis; (6) patients with periodontal disease; (7) patients with a history of orthodontic treatment; and (8) patients with morphologic anomalies such as fusion, gemination, concrescence, and dens in dente.

### Radiographic examination

Pre-treatment radiographs of each patient were examined. All radiographs were captured using Orthophos XG machine (Densplay Sirona, Germany). The following canine position measurements were made from the panoramic radiographs: (1) The horizontal position of the IMC relative to the adjacent teeth (S-Sector): IMC’s cusp tip overlap of the adjacent lateral incisor root was determined according to the method described by Ericson and Kurol [5]. (2) The angle of the IMC with the midline (α-angle): Angle of the long axis of the IMC with relative to the midline plane was determined as described by Ericson and Kurol [5]. (3) The distance from the IMC to the occlusal plane (d-distance): Distance from the cusp tip of the IMC to the occlusal plane was determined as described by Ericson and Kurol [5]. (4) The vertical height of the IMC relative to the adjacent lateral tooth (V): Vertical height of the IMC’s cusp tip to the adjacent lateral incisor root was determined as described by Counihan et al. [40]. (5) The apex position of the IMC (A): Apex position of the IMC was determined as described by Counihan et al. [40].

All the examinations and measurements were performed on a 27-inch colored LCD screen with 3.7 MP, 68 cm, and 2560 × 1440 resolution (RadiForce MX270W, Eizo Nanao Corporation, Ishikawa, Japan) via the software Fiji ImageJ (National Institutes of Health, Bethesda, MD, USA) under subdued lighting. The measurements were repeated one week later by Y.Ö.G. The arithmetic means of the distance and angle values obtained the previous week and the last values obtained were recorded.

**Fig. 1:**
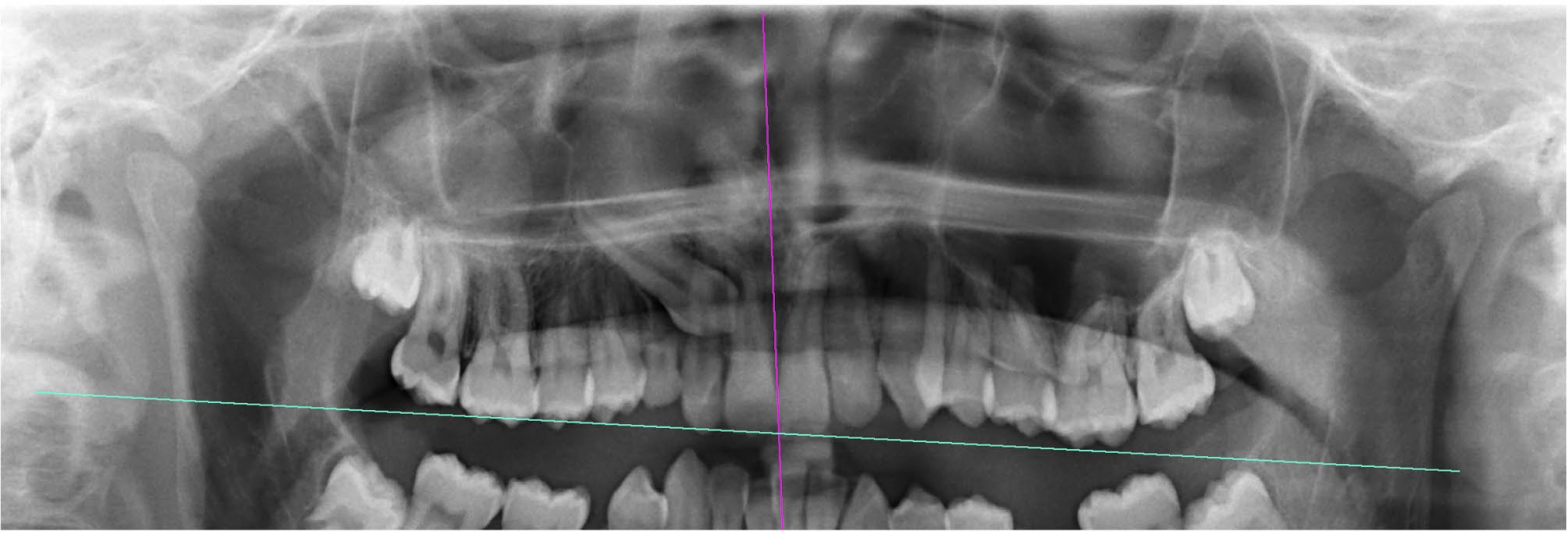
The midline plane [5] (purple line) passing through the anterior nasal spine (ANS) and the contact of the central teeth (ANS-1) and the occlusal plane [33] (blue line) passing through the mesiobuccal cusp of the upper first molar and the incisal edge of the upper central tooth (6 MB-1)

The reference planes are explained in Fig. 1. The occlusal plane is drawn according to the side of the impacted canine tooth. In bilateral cases, the occlusal plane was drawn separately for both canines (Fig. 1).

### Quantitative measurements

**Fig. 2:**
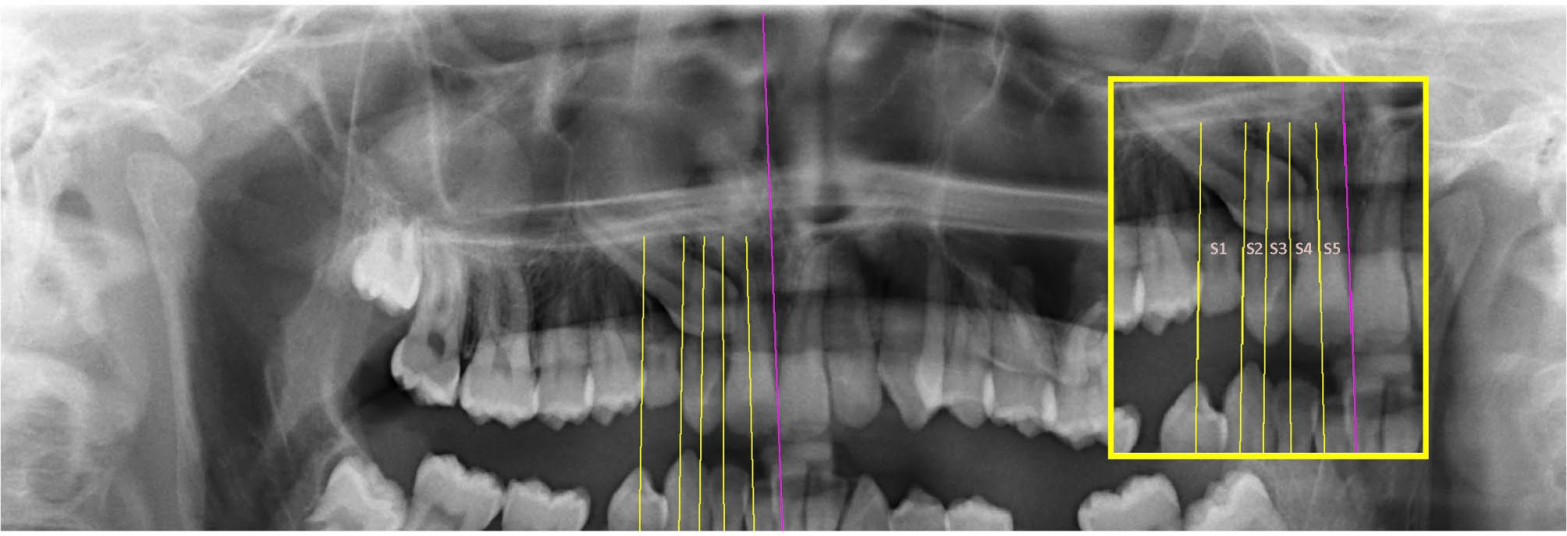
Sector 1: The zone between the axis passing through the mesial edge of the maxillary first premolar and the axis passing through the distal edge of the maxillary lateral tooth. Sector 2: The zone between the axis passing through the distal edge of the maxillary lateral tooth and the axis bisecting the long axis of the maxillary lateral tooth. Sector 3: The zone between the axis bisecting the long axis of the maxillary lateral tooth and the axis passing through the distal edge of the maxillary central tooth. Sector 4: The zone between the axis passing through the distal edge of the maxillary central tooth and the axis bisecting the long axis of the maxillary central tooth. Sector 5: The zone between the axis bisecting the long axis of the maxillary central tooth and the midline plane

**Fig. 3:**
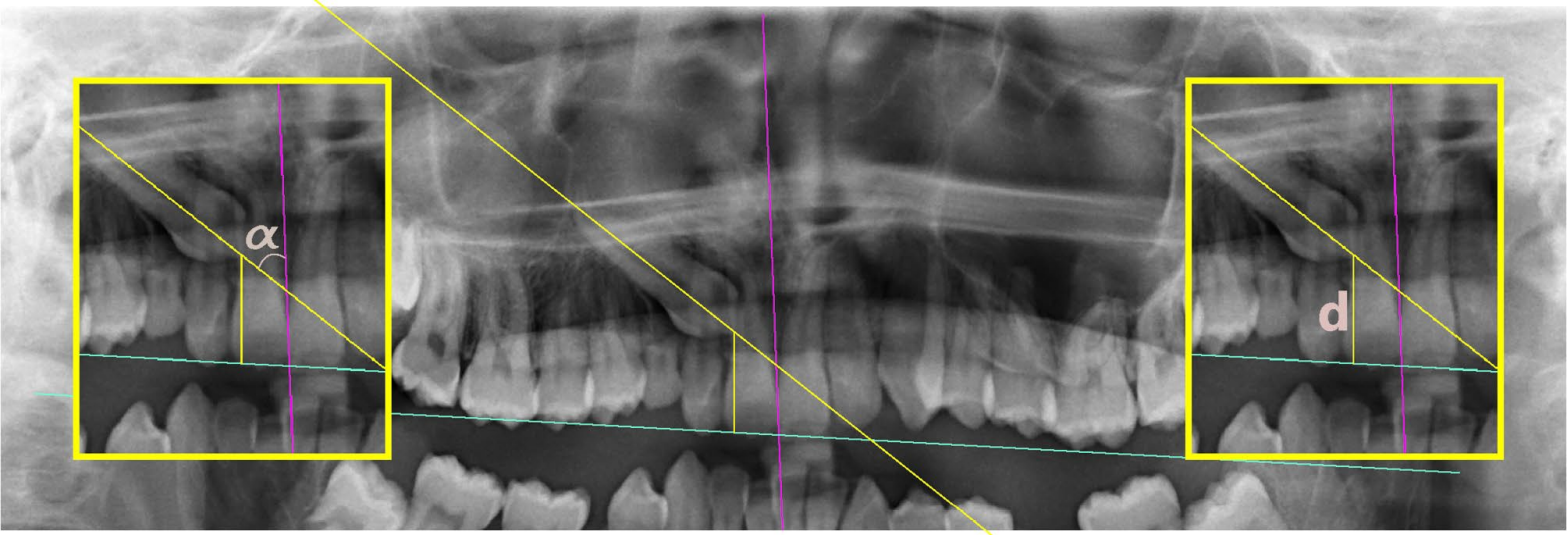
α is the angle between the axis passing through the long axis of the IMC and the midline plane. The d-distance is the length perpendicular to the cusp tip of the IMC to the occlusal plane

**Fig. 4:**
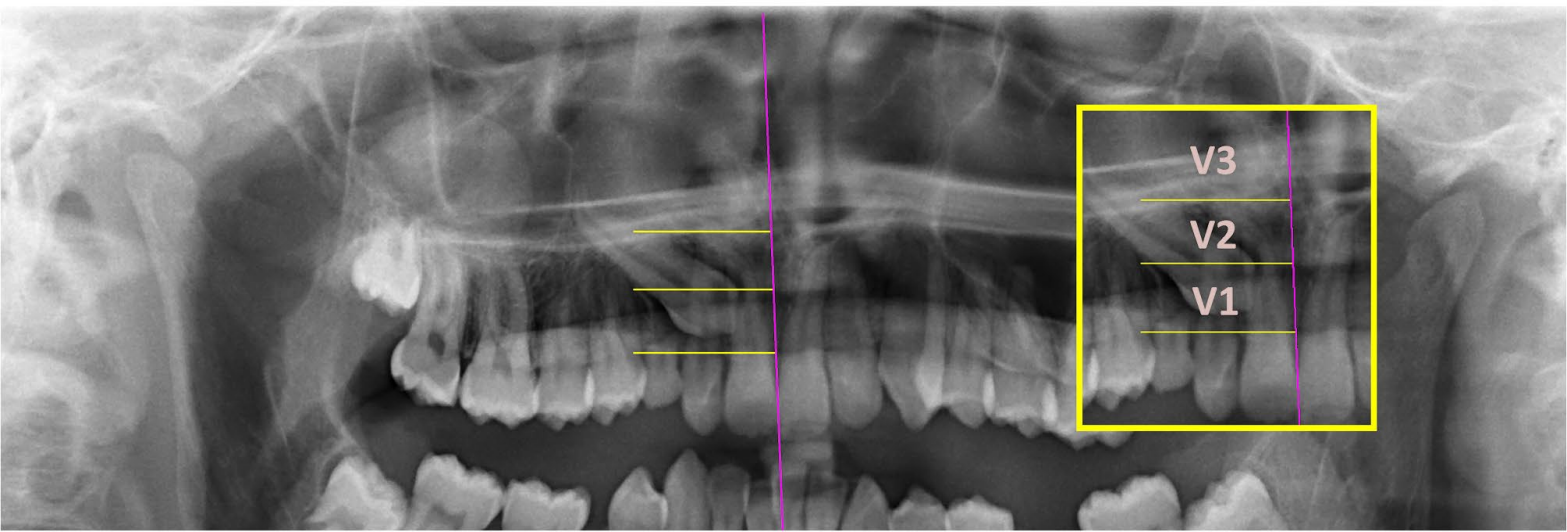
V1: The zone where the cusp tip of the IMC is between the cementoenamel junction of the adjacent lateral tooth and the axis bisecting the root. V2: The zone where the cusp tip of the IMC is between the axis bisecting the adjacent lateral tooth and the apex. V3: The zone where the cusp tip of the IMC is above the apex of the adjacent lateral tooth

**Fig. 5:**
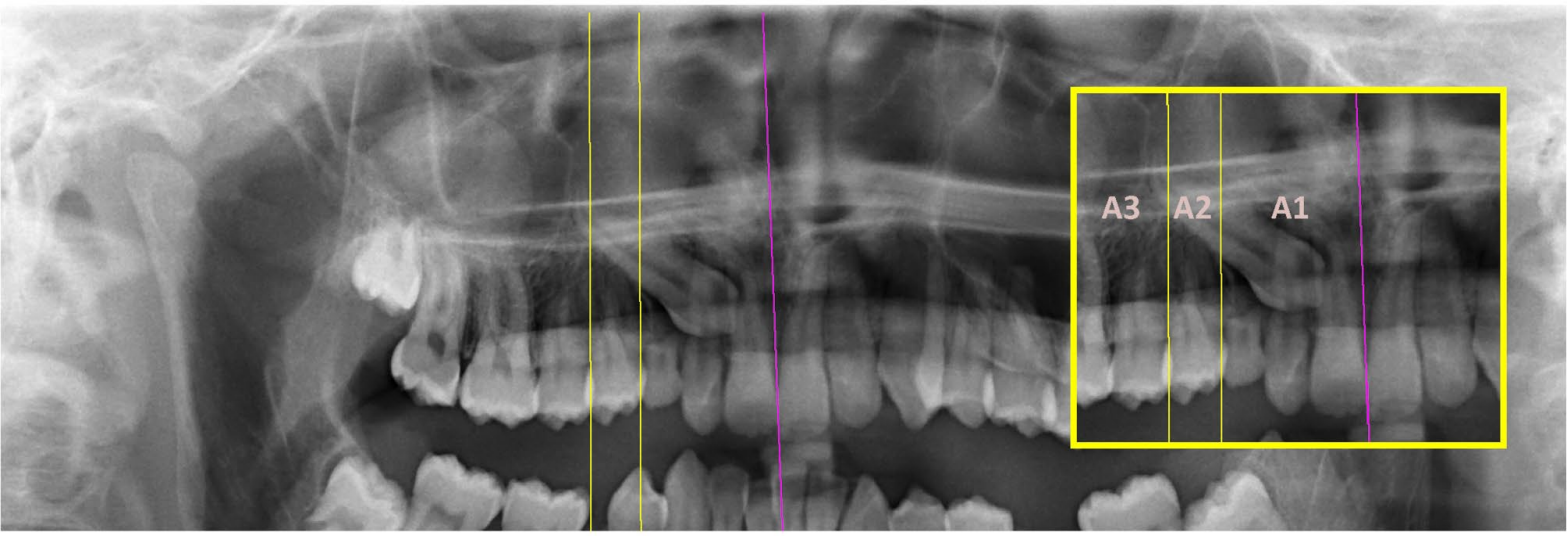
A1: The zone where the apex of the IMC is between the midline plane and the plane passing through the mesial edge of the first premolar. A2: The zone where the apex of the IMC is between the plane passing through the contact of the first and second premolars, and the plane passing through the mesial edge of the first premolar. A3: The zone where the apex of the IMC is distal to the plane passing through the distal edge of the second premolar

Quantitative measurements made to determine the position of the IMC were defined in terms of 5 parameters (Figs. 2, 3, 4 and 5). Besides, traction duration and age at the start of traction were recorded in Table 1.

### Qualitative parameters

In addition to the quantitative parameters mentioned above, qualitative parameters such as sex, treatment success, and impaction site were also evaluated. Their definitions and abbreviations are provided collectively in Table 1. For buccopalatal position prediction, the magnification and vertical restriction methods were used in the panoramic radiograph [41].

**Table 1:** Definitions of parameters

Parameter	Unit	Definition
Age	Month	Chronological expression in months of the time when traction of the IMC was started.
Traction duration	Day	Expression of the time in days from the time the IMC is surgically exposed and force is applied until the IMC appears in the mouth [42].
Treatment success	-	Cases are divided into success and failure.
Sex	-	The distribution of screened cases by sex was investigated.
Tooth number	-	The distribution of bilateral and unilateral cases was investigated.
Buccopalatal position	-	Buccopalatal positions of the IMCs were recorded as ‘buccal‘ and ‘palatal’.

### Treatment progress

In all the cases, a preadjusted straight wire 0.022′′×0.028′′ slot MBT brackets was used. The overall combined treatment was divided into 3 phases:

1. Levelling & alignment and creating space for IMC in the dental arch.
2. Surgical exposure (closed exposure technique) and orthodontic traction were applied at the 0.021′′×0.025′′ stainless steel wire phase. A handmade chain was connected to the attached device on the impacted tooth, and traction was then used to guide the impacted canine toward the center of the alveolar ridge. When the appropriate vertical position of the orthodontically erupted IMC was obtained, it was moved onto the arch with a Kilroy spring.
3. The final orthodontic treatment aligned the canine in the maxillary arch.

Successful treatment was defined as achieving fully functional eruption of the canine. Failure of treatment was defined as the need for extraction of the IMC. The clinician tried traction for a certain period of time and extracted the tooth when it could not be moved. The cause of failure was ankylosis in all failure cases.

### Statistical analysis

The data were analyzed with IBM SPSS V23 (Chicago, IL, USA). The intraclass correlation coefficient was used to evaluate the relationship between two different measurements, and binary logistic regression was used to determine the factors affecting treatment success. Linear regression was conducted to determine the relationships between traction duration and other parameters. To create a classification system to determine the prognosis of IMCs, a cutoff value was calculated using the α-angle and d-distance. Receiver operating characteristic (ROC) curve analysis was used to determine the cutoff value. The significance level was set at *p* < 0.05.

## Results

A total of 134 IMCs in 121 patients were evaluated. Among these patients, 13 had bilateral IMCs, and 108 had unilateral IMCs. Thirty-four (28.1%) of the patients were male, and 87 (71.9%) were female. The mean age of the females was 16.2 years, and the mean age of the males was 16.21 years. The overall mean age was 16.2 years.

### Agreement between measurements

One week after the first measurement, the second measurement was performed by the same researcher (Y.Ö.G.). The agreement between the first and second measurement values was very high (Table 2). The analysis was continued by averaging the α-angle and d-distance.

**Table 2:** Intraexaminer reliability

	Mean ± SD	Median (min–max)	ICC (95% CI)	*p*
**α-angle**				
1st measurement	36.34 ± 15.64	35.4(7.6–91.6)	0.999(0.999-1.000)	**< 0.001**
2nd measurement	36.38 ± 15.61	35.5(6.9–91.7)		
**d-distance**				
1st measurement	20.68 ± 5.41	20.5(9.7–36.8)	0.995(0.993–0.997)	**< 0.001**
2nd measurement	20.69 ± 5.42	20.4(9.9–36.6)		

ICC: Intraclass correlation coefficient; CI: Confidence interval; SD: Standard deviation; *p* < 0.001 indicates excellent agreement

### Results related to the buccopalatal position

The statistical findings of Pearson’s chi-square test for the distribution of the sector of IMCs according to their buccopalatal position are presented below. When we examined whether the buccopalatal position of the IMC and the sector were related, a statistically significant relationship was found (*p* < 0.001) (Table 3). Buccally impacted maxillary canine teeth are mostly located in sectors 1–2. Palatally impacted maxillary canine teeth are mostly located in sectors 3-4-5.

**Table 3:** Relationship between buccopalatal position and sector*

	Palatal (n/%)	Buccal (n/%)	Total (n/%)	Test Statistics	p
Sector 1	2 (2)	19 (54.3)	21 (15.7)		
Sector 2	13 (13.1)	13 (37.1)	26 (19.4)		
Sector 3	34 (34.3)	3 (8.6)	37 (27.6)	76.653	**< 0.001**
Sector 4	28 (28.3)	0 (0)	28 (20.9)		
Sector 5	22 (22.2)	0 (0)	22 (16.4)		

*Pearson’s chi-square test

### Evaluation of risk factors affecting treatment success

Logistic regression was applied to evaluate the effects of the parameters measured in our study on treatment success (Table 4).

Age, d-distance, and α-angle were found to be risk factors affecting treatment success. The risk of failure increased 1.015-fold with increasing age (*p* = 0.003). As the α-angle increased, the risk of failure increased 1.074-fold (*p* = 0.002). Similarly, the risk of failure increased 1.206-fold as the d-distance increased (*p* = 0.001).

In the multivariate regression, only age was a risk factor. The odds ratio (OR) value for age was 1.022 (*p* = 0.002). The other variables were not identified as risk factors.

**Table 4:** Examination of risk factors affecting treatment success*

	Treatment	outcome		Univariate		Multivariate	
	Successful	Failure	Total	OR (95% CI)	*p*	OR (95% CI)	*p*
**Vertical height of the IMC** **relative to the adjacent tooth**							
V1	111 (93.3)	8 (6.7)	119 (88.8)	Reference		Reference	
V2	12 (80)	3 (20)	15 (11.2)	3.469 (0.810:14.853)	0.094	0.345 (0.02–5.9)	0.462
**Sex**							
Female	90 (91.8)	8 (8.2)	98 (73.1)	Reference		Reference	
Male	33 (91.7)	3 (8.3)	36 (26.9)	1.023 (0.256:4.088)	0.975	0.726 (0.106–4.961)	0.744
**Tooth number**							
13	65 (89)	8 (11)	73 (54.5)	2.379 (0.603:9.395)	0.216	0.178 (0.022–1.467)	0.109
23	58 (95.1)	3 (4.9)	61 (45.5)	Reference		Reference	
**Type of impaction**							
Unilateral	99 (91.7)	9 (8.3)	108 (80.6)	1.091 (0.221:5.380)	0.915	0.602 (0.074–4.892)	0.635
Bilateral	24 (92.3)	2 (7.7)	26 (19.4)	Reference		Reference	
**Age (months) (mean ± SD)**	189 ± 38.4	248.3 ± 85.7	193.8 ± 46.6	1.015 (1.005:1.026)	**0.003**	1.022 (1.008–1.036)	**0.002**
**α-angle** (°) **(mean ± SD)**	34.9 ± 14.5	52.5 ± 19	36.4 ± 15.6	1.074 (1.027:1.123)	**0.002**	1.06 (0.978–1.148)	0.156
**d-distance (mm) (mean ± SD)**	20.2 ± 5.2	26.4 ± 5	20.7 ± 5.4	1.206 (1.079:1.348)	**0.001**	1.239 (0.983–1.562)	0.070
**Traction duration (day) (mean ± SD)**	160.8 ± 106	198.2 ± 116	163.9 ± 106.9	1.003 (0.998:1.008)	0.271	0.997 (0.988–1.006)	0.495
**Sector**							
Sectors 1-2-3	78 (92.9)	6 (7.1)	84 (62.7)	Reference			
Sectors 4–5	45 (90)	5 (10)	50 (37.3)	1.444 (0.417–5.003)	0.562	0.587 (0.086–4.009)	0.587
**Apex position of the IMC**							
A1-2	92 (93.9)	6 (6.1)	98 (73.1)	Reference			
A3	31 (86.1)	5 (13.9)	36 (26.9)	2.473 (0.705–8.672)	0.157	1.759 (0.273–11.347)	0.552

*Logistic Regression Analysis; OR: Odds ratio; CI: Confidence interval; SD: Standart deviation

**Fig. 6:**
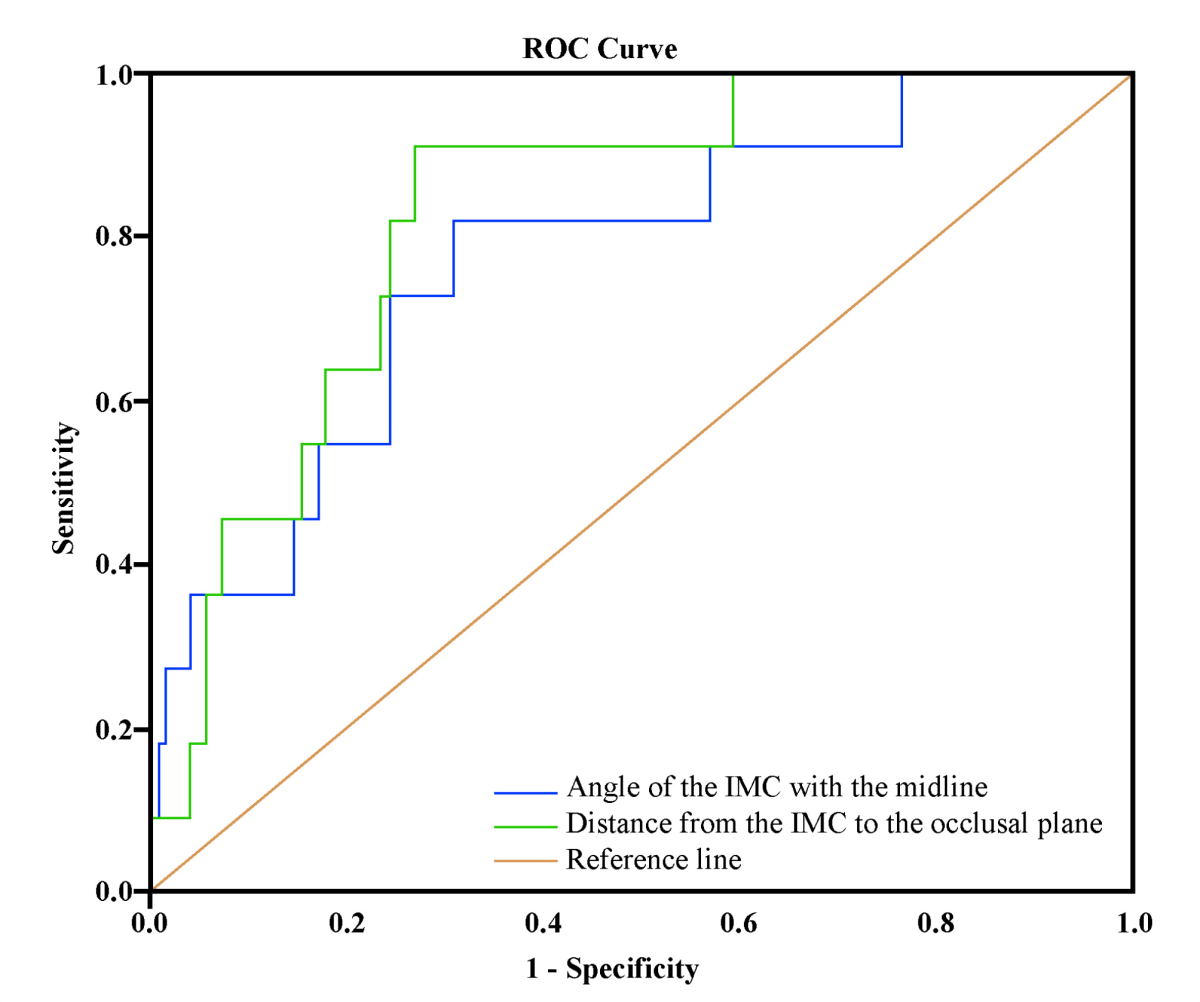
ROC analysis results

ROC analysis was performed to find the limit values of the α-angle and the d-distance, which were identified as risk factors for treatment success (Table 5-Fig. 6).

The area under the ROC curve (AUC) for the α-angle was 77.2% (*p* = 0.003). When the α-angle was 44.3° or above, it was determined that success would be at risk. The sensitivity and specificity for this cutoff value were 81.8% and 69.11%, respectively.

Similarly, the AUC value for the d-distance was 82.7% (*p* < 0.001), and a risk of failure was determined to occur at a distance of 22.3 mm or greater. The sensitivity and specificity for this cutoff value were 90.91% and 73.17%, respectively.

**Table 5:** ROC analysis results for angle and distance values for treatment success

	Cutoff value	AUC (95% CI)	*p*	Sensitivity	Specificity		
**α-angle**	≥ 44.295	0.772 (0.627:0.916)	**0.003**	81.82	69.11		
**d-distance**	≥ 22.295	0.827 (0.722:0.932)	**< 0.001**	90.91	73.17		

ROC: Receiver operating characteristic; AUC: Area under the ROC curve; CI: Confidence interval

### Evaluation of factors affecting traction duration

Linear regression analysis was performed to identify the factors affecting traction duration (Table 6). As the d-distance increased by 1 mm, the traction duration increased by 8.4 days (*p* < 0.001). When the sector variable was added to the model as a dummy variable, the traction duration increased by 73 days in sector 4 and by 99.5 days in sector 5 (*p* < 0.001). As age increased, traction duration decreased by 0.2 days (*p* = 0.049). With the model created, 82% of the traction duration is explained.

**Table 6:** Results of linear regression analysis of traction duration and other parameters

	β^1^ (95% CI)	SH	β^2^	t	p	Zero	Partial	Part
d-distance	8.4	1.013		8.325	**< 0.001**			
Sector 5	99.5	20.354	0.206	4.890	**< 0.001**	0.515	0.394	0.179
Sector 4	73	18.019	0.171	4.049	**< 0.001**	0.486	0.335	0.148
Age	-0.2	0.108	- 0.218	- 1.992	**0.049**	0.814	-0.172	-0.073

F(4,130) = 154.009; *p* < 0.001; Adj. R^2^ = 0.820; SH = 82.832; [1]: Unstandardized coefficient; [2]: Standardized coefficient; Durbin–Watson = 1.902

## Discussion

The first step in the diagnosis and treatment planning of IMCs is to determine the possibility of eruption of the impacted tooth. Clinicians need to estimate the duration of treatment and prognosis as close to reality as possible when informing patients during the initial examination before further evaluation. The drawings that can be made with a simple program on the panoramic radiograph and the limit values to be used here will enable the patient to see the position of the impacted tooth more clearly and will ensure trust between the patient and the clinician. Therefore, it is necessary to clearly ascertain the position of the IMC.

Many studies on IMCs have been reported in the literature. These studies have focused mostly on predicting whether IMCs will erupt normally during mixed dentition via panoramic radiographs [5, 7, 17, 43–45], evaluating the effectiveness of panoramic radiography results compared with cone beam computerized tomography (CBCT) in the analysis of IMCs [46–48], and diagnosing resorption of adjacent teeth [28]. The relationships between radiographic findings, traction duration, and treatment success, which affect the prognosis of IMCs requiring orthodontic treatment and surgical intervention, are unclear.

In the present study, the radiographic features of IMCs treated in an orthodontic clinic were classified, and the relationships between these radiographic features and the traction duration required for alignment of IMCs and the factors affecting treatment success were investigated. We also examined which prediction method is more effective in terms of prognosis and whether measurements have an effect on traction duration. Unlike many previous studies [25, 27, 36] in which factors affecting treatment duration were investigated, IMCs whose orthodontic traction failed were also included in the evaluation.

The sector of the IMCs determined on panoramic radiographs was used to evaluate their buccopalatal position. Jung et al. [49] reported that IMCs in sectors 1-2-3 on panoramic radiographs were mostly impacted buccally, those in sector 4 were mostly impacted in the middle of the alveolus, and those in sector 5 were mostly impacted palatally. Baidas et al. [50] reported that buccally, IMCs were mostly located in sector 1 and that palatally, IMCs were mostly located in sectors 3–4. Alfaleh et al. [29] reported that buccally, IMCs were mostly located in sectors 1–2 and that palatally, IMCs were mostly located in sectors 3–4. In our study, in accordance with the results of previous research, the IMCs in sectors 1–2 were mostly buccal, and those in sectors 3-4-5 were mostly palatal (*p* < 0.001). According to guidance theory [2], during tooth development, maxillary permanent canines erupt along the distal aspect of the root of the adjacent lateral tooth. If this guidance is not absent, the canine will erupt palatally because of the V-shaped structure of the maxilla. Thus, the sector of IMCs increases. Since only a single panoramic radiograph was used in the study, the buccopalatal position of the IMC were estimated. Magnification and vertical restriction were based on the research by Chaushu et al. [41]. This method is reliable when the IMC is located in the coronal and middle zones of the adjacent tooth but is not reliable for teeth in the apical zone. Our study utilized a retrospective cross-sectional design and analyzed panoramic radiographs of individuals with IMC treated by different clinicians in the same clinic between 2010 and 2023. Therefore, radiographs were not captured under standard conditions (radiographs were captured with the same machine, but some of them had slightly different exposure values). CBCT [48] scanning or exposure of the impacted tooth, which is the most ideal method for determining the buccopalatal position of the IMC within the alveolus, cannot be used.

Koçyiğit et al. [38] reported that the sector of IMC did not affect the treatment success, whereas Motamedi et al. [51] reported that the prognosis worsened if the cusp tip of the IMC exceeded half of the root of the adjacent lateral tooth. Grisar et al. [19] associated more mesial sectors with inferior treatment outcomes. In our study, the horizontal position of the IMC relative to the adjacent teeth, which we divided into 5 sectors, was grouped into two groups to apply the statistical method. Sectors 1-2-3 were considered one group, and sectors 4–5 were considered the other group. According to the results of our study, there was no significant difference between the two groups in terms of treatment success (*p* = 0.587). No difference was explained by the fact that traction of the IMC is a vertical movement, whereas this measurement was horizontal. In addition, the horizontal position of the IMC relative to the adjacent teeth may be related to root resorption rather than treatment success since it is a definition that expresses the relationship with adjacent teeth [52].

In some studies [36, 53], a significant relationship was not found between the sector of the IMC and traction duration. Dubovská et al. [37] reported that the sector of the IMC was significantly related to the total treatment time. Bazargani et al. [54] reported that the mean treatment time for IMCs in sectors 1–2 was 17 months, increased by 2.6 months in sector 3, and increased by 7.6 months in sectors 4–5. Fleming et al. [55] reported a significant relationship between the sector of IMC and total treatment time. Zuccati et al. [56] divided the position of the IMC into 5 sectors, and to obtain a statistically significant result, they considered sectors 1-2-3 as one group and sectors 4–5 as a separate group, as in our study, and they reported a significant relationship between the 2 groups in terms of traction times. Additionally, some studies [19, 24, 42, 57–61] reported a positive relationship between sector position and traction and/or treatment duration. In our study, no significant correlation was found between the IMC sector and traction duration in sectors 1-2-3, whereas a significant correlation was found in sectors 4–5. The traction duration increases by 73 days when the IMC is located in sector 4 and increases by 99.5 days when the IMC is located in sector 5 (*p* < 0.001). This may be because the IMC is located in a more vertical position in sectors 1-2-3 and does not affect the traction duration, whereas in sectors 4–5, the cusp tip is vertically further away from the oral cavity owing to the anatomy of the premaxilla.

According to some researchers [19, 51], if the angle of the IMC with the midline increases, the possibility of treatment success decreases. In another study [38], there was no significant relationship between the angle of the IMC with the midline and treatment success. In our study, there was a significant relationship between the angle of the IMC with the midline and treatment success. An angle greater than 44.3° was a significant risk factor for treatment success (*p* < 0.002). Since the increase in the angle of the IMC with the midline requires more correction in the long axis of the tooth, the possibility of complications during traction may increase [33, 62, 63], constituting a risk factor for treatment success.

According to some studies [19, 23, 27, 42, 54, 56, 58–60], there is a significant direct relationship between the angle of the IMC with the midline and the treatment duration. In contrast, there are studies in which it is stated that there is no such significant relationship [24, 38, 53, 55]. In our study, no significant relationship was found between the angle of the IMC with the midline and the traction duration. The factors associated with th traction duration were thought to be more related to the vertical position of the cusp tip in the bone.

In a study [64] in which the vertical height of the IMC relative to the adjacent lateral tooth on treatment success was examined, the prognosis was poor for IMCs close to the level of the root tip. In another study [65], the effect of vertical height of the IMC on treatment prognosis was estimated, and the prognosis was classified as poor, average or good. Stivaros and Mandall [66] investigated the factors affecting the orthodontist’s decision to expose or remove the IMC and reported that the vertical height relative to the adjacent tooth was not a factor affecting their decision. In our study, the vertical height of the IMC relative to the adjacent lateral tooth was not significantly related to treatment success or traction duration (*p* = 0.462). This result may have occurred because there was no IMC at the ‘V3 height’ in our study.

In a study [38] in which the relationship between the distance from the IMC to the occlusal plane and treatment success was investigated, no statistically significant relationship was found. Grisar et al. [19] associated a greater vertical position with inferior treatment outcomes. In our study, a significant relationship was obtained, and the risk of failure was greater at distances of 22.3 mm and above (*p* < 0.001). Since the distance from the IMC to the occlusal plane has a direct effect on the traction duration, an increase in this distance will lead to more side effects [33, 62, 63], such as resorption and ankylosis in the tooth, that are forced for longer periods. Since this may decrease the prognosis of traction, it is clinically important to obtain this result.

In addition to the studies [19, 36, 42, 53, 54, 56, 58–60] in which a direct correlation between the distance from the IMC to the occlusal plane and the traction duration (or treatment time) was reported, there are also studies [24, 55] in which there was no significant relationship between them. In our study, a significant correlation was obtained between the distance from the IMC to the occlusal plane and traction duration (*p* < 0.001). When the distance increased by 1 mm, the traction time increased by 8.4 days. Therefore, the greater the eruption path distance of the impacted tooth is, the longer the traction duration.

Amuk et al. [21] stated that the presence of a root–cortex relationship also prolonged treatment duration for approximately 3 months. Rodríguez-Cárdenas et al. [67] although the roots of palatally and buccally impacted canine teeth moved to different degrees during treatment, a significant change in traction time was not observed. In our study, no significant correlation was found between the apex position of the IMC and either treatment success or traction duration (*p* = 0.552). This result is explained by the lack of sufficient cases at the A1 and A2 positions.

In a study [68] in which the effects of patient age on treatment success and duration were compared between adolescents and adults, there was no statistically significant difference in treatment success due to the insufficient number of failed cases, but the success rate was 100% in adolescents and 69.5% in adults. In the same study, there was no significant difference between the two groups in terms of total treatment time, but the time required for successful traction of an IMC was 2 times longer in the adults than in the adolescents, and this result was statistically significant. In our study, in accordance with the literature [39, 69], patient age affected treatment success. Treatment success decreased significantly with increasing age (*p* < 0.003). Bone density and the possibility of damage to periodontal tissues [70] increase with increasing age, which explains this result. Zuccati et al. [56] reported that the number of appointments required for alignment of IMCs increased with age in a study including 87 individuals. Although many studies [23, 55, 57, 60, 61] have reported that age has no effect on the traction duration, our findings regarding age contrast with previously published results. However, some studies [25, 42, 53] have reported that the duration of treatment decreases with increasing age. With respect to the effect of age on the traction duration, the findings of our study revealed that the traction duration decreased with increasing age (*p* = 0.049).

In our study, we ignored the skeletal classification of the patients. Although the type of skeletal malocclusion affects treatment time, there is no conclusive evidence on whether IMCs affect traction duration and treatment success [24, 71].

One factor affecting the treatment success and traction duration of IMCs is morphological features; in particular, dilaceration is frequently observed in IMCs [25]. In some studies [72, 73], dilaceration was more common in IMCs than in nonimpacted maxillary canines. Therefore, we included the mild forms of dilaceration observed on radiographs, which can be considered ‘natural’ for IMCs.

CBCT images of most of the patients could not be included in the study because we could not access them during the archive search. Distortions, magnifications and superimposition of adjacent structures in panoramic radiography reduce its effectiveness in the diagnosis of IMCs. Three-dimensional imaging provides more precise information about the relationship between the IMC and the adjacent structures in 3 planes, more precise information on the resorption of the IMC and adjacent teeth, and more precise information on all issues, from the precise diagnosis of the localization to the choice of surgical technique [74]. However, the use of CBCT is still limited due to its disadvantages, such as its high cost and radiation dose and the need for expertise for correct interpretation.

## Conclusion

**Fig. 7:**
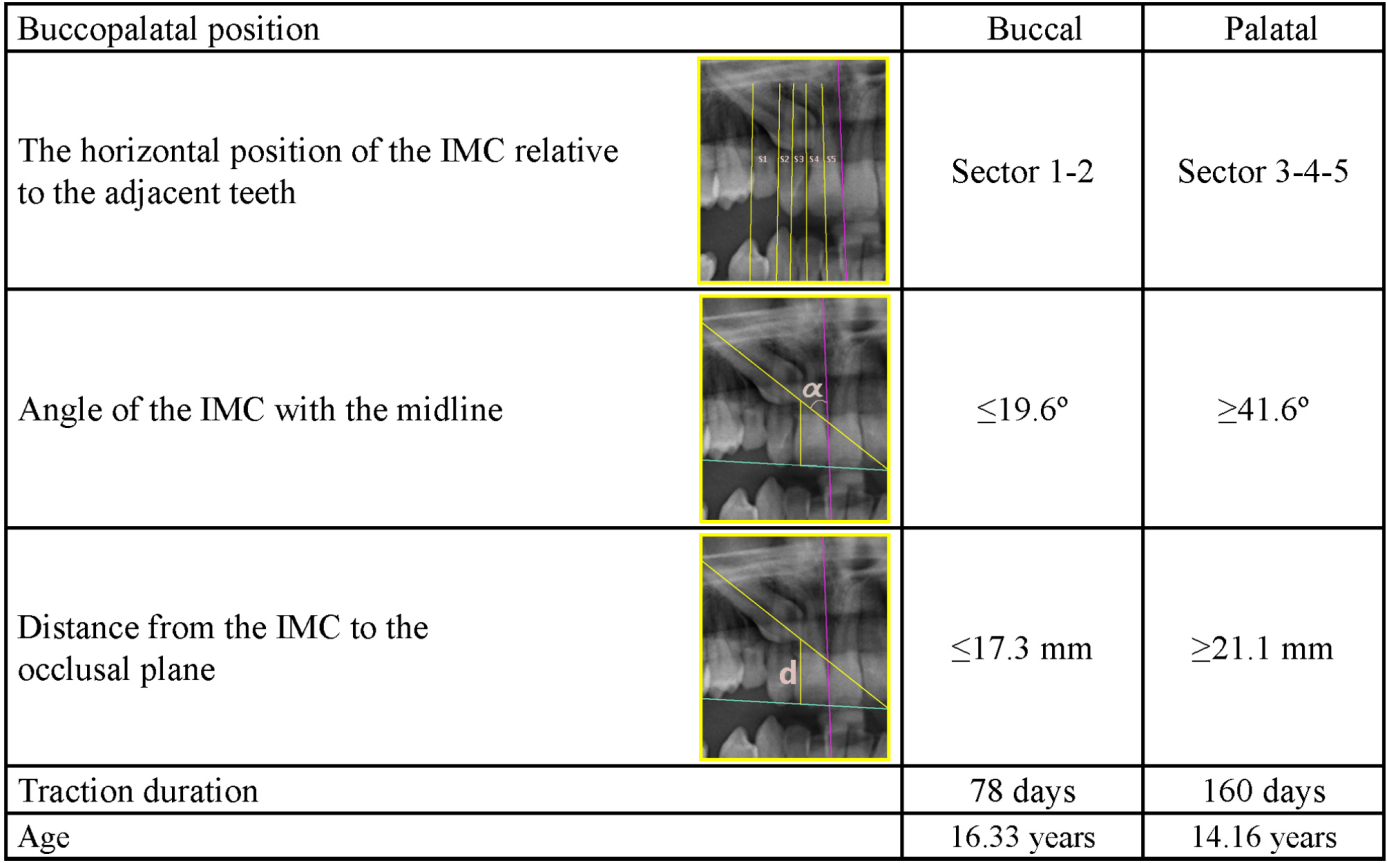
Prediction of buccopalatal position

**Fig. 8:**
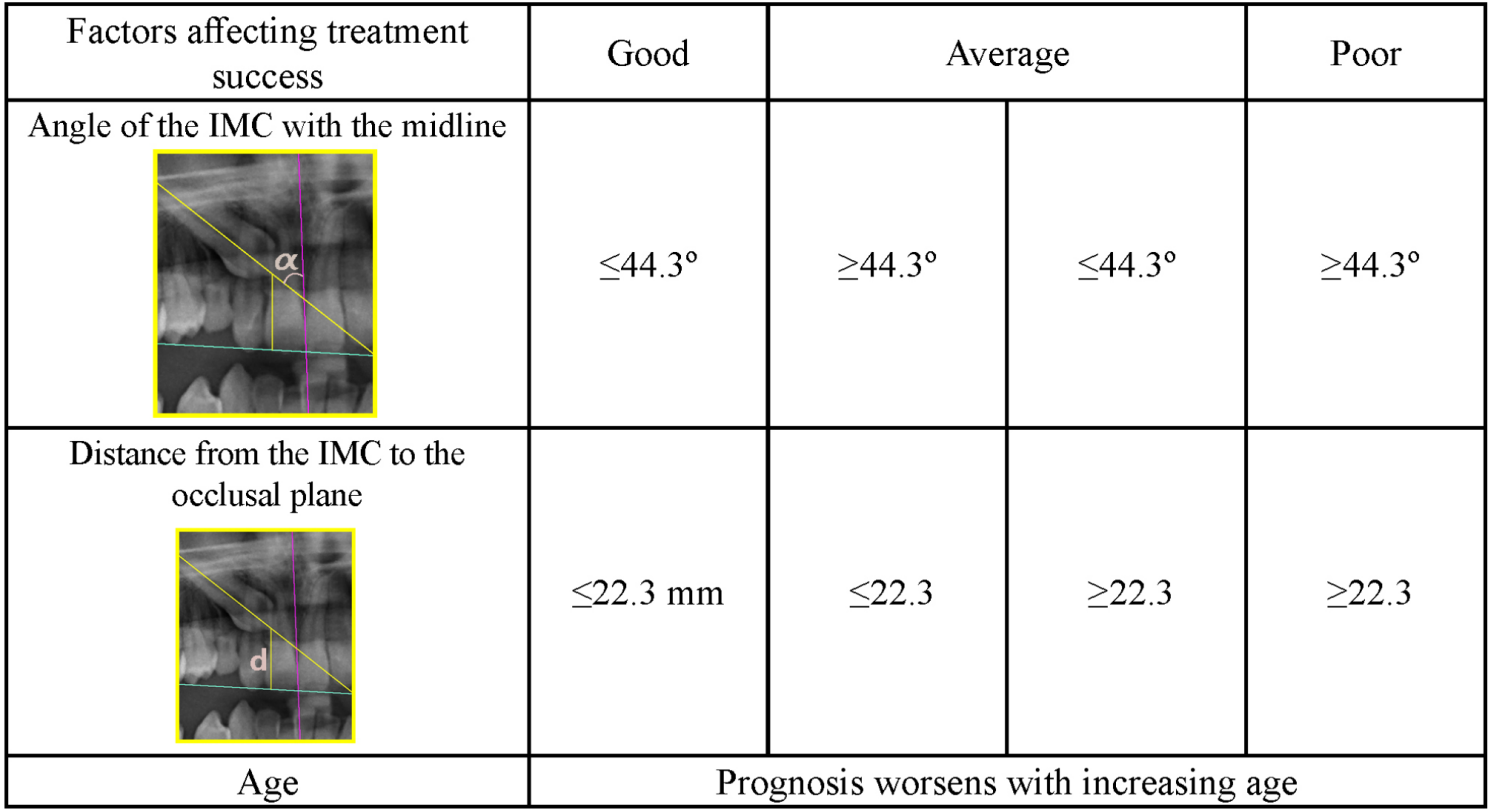
Factors affecting treatment success

**Fig. 9:**
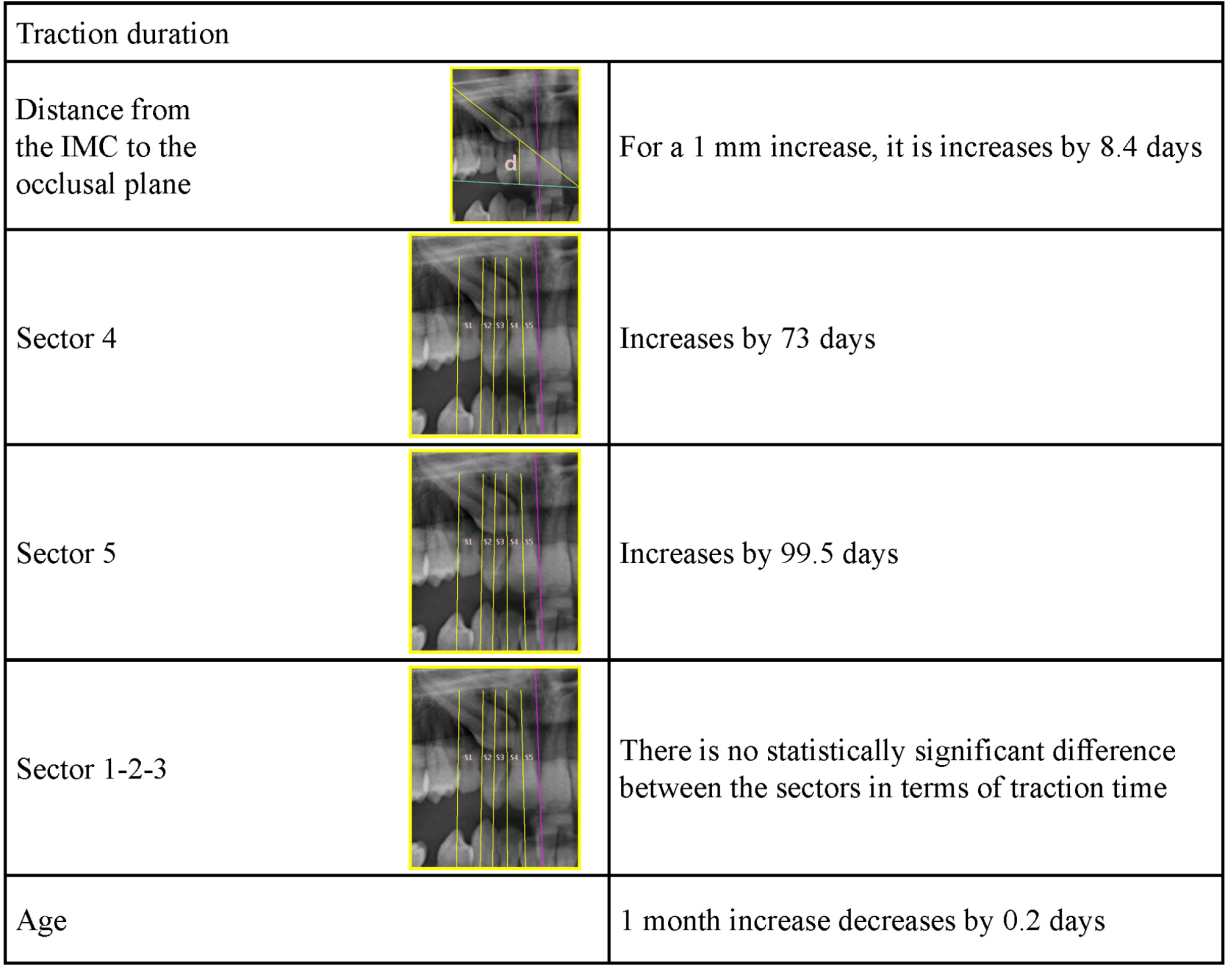
Prediction of traction duration

The findings of our study are summarized as follows: (Figures 7, 8 and 9)

1. The alternative (H1) hypothesis of our study was that ‘The success of orthodontic treatment of IMCs and the traction duration depend on the measurement parameters on panoramic radiographs’. According to the results obtained, the alternative hypothesis was accepted.
2. A buccopalatal position prediction table (Fig. 7) was created for IMCs.
3. Age, the distance from the IMC to the occlusal plane, and the angle of the IMC with the midline were found to affect treatment success and were associated with a prognostic classification (Fig. 8).

4) The estimated traction or mean treatment duration was calculated from the distance from the IMC to the occlusal plane, the horizontal position of the IMC relative to the adjacent teeth and age (Fig. 9).
5) For prognosis prediction, the distance from the IMC to the occlusal plane is a more decisive parameter than the vertical height of the IMC relative to the adjacent lateral tooth.

## Data Availability

First measurement: https://docs.google.com/spreadsheets/d/1oekjpTw_uLfXIYFw47a806z1UwprDKLD/edit?usp=sharing&ouid=101560659499522509171&rtpof=true&sd=true. Second measurement: https://docs.google.com/spreadsheets/d/1-QpZuL9fr7qdzh85-0mP_bzelYUyX7ly/edit?usp=sharing&ouid=101560659499522509171&rtpof=true&sd=true. Average values: https://docs.google.com/spreadsheets/d/1jlzVyPOayL9BvETUizPH87ajCy63Ak4E/edit?usp=sharing&ouid=101560659499522509171&rtpof=true&sd=true.

## Abbreviations

IMC: Impacted maxillary canine
ROC: Receiver operating characteristic
H1: Alternative hypothesis
AUC: Area under the ROC curve
OR: Odds ratio
CBCT: Cone beam computed tomography
